# Novel Fault Diagnosis Technology Based on Integrated Spectral Kurtosis for Gearboxes

**DOI:** 10.3390/s26072185

**Published:** 2026-04-01

**Authors:** Len Gelman, Rami Kerrouche, Abdulmumeen Onimisi Abdullahi

**Affiliations:** Department of Engineering, School of Computing and Engineering, The University of Huddersfield, Huddersfield HD1 3DH, UK; r.kerrouche@hud.ac.uk (R.K.);

**Keywords:** fault diagnosis, motor current signature analysis, vibration, spectral kurtosis, gearbox, condition monitoring, signal processing

## Abstract

This paper proposes a novel integrated spectral kurtosis (ISK) technology, which is a new conceptualization for fault diagnosis, and compares it with conventional spectral kurtosis technology. The vibration signals from a gearbox are processed by time synchronous averaging (TSA) and analysed using the spectral kurtosis (SK). The ISK feature is estimated across the entire frequency domain, while the envelope is obtained through SK-based filtering and a Hilbert demodulation. The ISK technology demonstrates the ability to distinguish between healthy and defected gearbox cases, achieving a total probability of correct diagnosis (TPCD) of 91.5% for pinions and 96.1% for gears, whereas the SK-based squared envelope technology provides a limited diagnosis effectiveness, with a maximum TPCD of 80%. The motor current signals are also analysed through harmonic amplitude tracking within the current spectrum. A comparison of the ISK and motor current technologies is also made, showing that the motor current technology reaches a maximum of 90% TPCD for gears, which remains lower than the TPCD for the ISK technology.

## 1. Introduction

Gearboxes play a pivotal role in power transmission systems across diverse industrial domains, including wind turbines, automotive drivetrains, aerospace actuation systems, and heavy-duty manufacturing equipment [1]. Their primary function is to transmit torque and regulate the rotational speed between shafts. Due to their mechanical complexity and the continuous dynamic interaction of meshing teeth, gearboxes are highly susceptible to various fault modes, such as tooth breakage, pitting, spalling, and scuffing, particularly under fluctuating operational loads and environmental stresses [2,3,4]. The early-stage detection of these failure modes is imperative to prevent catastrophic breakdowns, optimize maintenance scheduling, and enhance system availability [5].

Among the various condition monitoring technologies, vibration condition monitoring has gained prominence as a reliable technology for gearbox fault diagnosis. Conventional time-domain features, such as the root mean square (RMS), crest factor, and kurtosis, are frequently employed to detect abnormal signal behaviour [6]. In parallel, frequency-domain features, particularly the gear mesh sidebands and harmonic content, have proven effective for identifying the modulation patterns associated with mechanical defects [7,8]. Nevertheless, these approaches often struggle in noisy, non-stationary environments and may fail to isolate weak transient signatures without additional signal enhancement steps [9].

To address these shortcomings, time synchronous averaging (TSA) has been used extensively to extract the deterministic vibration components linked to periodic gear meshing. TSA works by aligning and averaging the angularly resampled signals across multiple cycles, thereby suppressing the stochastic noise and reinforcing the periodic content [8,10]. Building upon this, residual signal extraction eliminates the dominant mesh components from the averaged signal, resulting in an enhanced waveform that emphasizes the low-amplitude, non-cyclic transients often linked to localized gear faults [11,12]. More recent approaches allow for TSA to be implemented without tachometric sensors, further enhancing its applicability in operational settings [13]. However, the effectiveness of TSA-based preprocessing ultimately depends on the effectiveness of the subsequent diagnostic feature extraction stage.

Beyond the second-order statistical metrics, higher-order signal analysis [14,15,16,17,18,19] offers superior effectiveness for fault diagnosis. The spectral kurtosis (SK) has also emerged as an effective technology for revealing the non-Gaussian transients masked within complex vibration signals [20,21]. Unlike classical kurtosis, which condenses the impulsiveness into a global scalar, the SK decomposes this information across frequency bins, producing a kurtogram that identifies the most informative spectral bands for demodulation [22,23]. SK-based technologies have been successfully applied to gearbox diagnostics [24,25,26,27,28,29,30,31,32,33,34,35,36] and typically culminate in a squared envelope (SE) analysis of the SK-selected narrowband. Despite these advances, the diagnostic capability of spectral kurtosis remains constrained by its reliance on the narrowband selection and the subsequent envelope-based processing.

However, despite its wide adoption, the SK-based squared envelope (SK-SE) technology suffers from critical limitations that restrict its use for diagnostics in practical gearbox monitoring. A squared envelope (SE), based on narrowband filtering and a Hilbert demodulation, performs energy averaging and cannot effectively separate the impulsive fault transients from the smooth structural modulations. Consequently, the dominant mesh harmonics often overshadow the fault-induced impacts, leading to poor diagnostic performance. In addition, the averaging inherent to time synchronous averaging (TSA) and residual extraction can further attenuate the weak, non-repetitive transients, reducing the effectiveness of SE-based features. Because the SK-SE operates within a narrowband, it also fails to capture the broadband or drifting impulsive energy that characterizes fault evolution. Phase misalignment further distorts transient localization, while its multi-step implementation adds computational overhead. These limitations define a clear research gap for a spectral kurtosis-based diagnostic technology capable of capturing impulsive fault information in a broadband and integrative manner. To overcome these limitations, this study introduces, for the first time worldwide, an integrated spectral kurtosis (ISK) technology, in which the classical spectral kurtosis is preserved, but its diagnostic usage is extended through a frequency-domain integration. The ISK technology differs from the existing SK-based technologies by its integrating of the spectral kurtosis values. Unlike kurtograms, protrugrams, and SK-based envelope analysis, which focus on identifying a limited number of optimal frequency bands, the proposed ISK technology is defined as the summation of the spectral kurtosis values exceeding a selected threshold across the full frequency range, yielding a single scalar diagnostic feature. To the best of the authors’ knowledge, such integration-based spectral kurtosis technology has not been previously reported for fault diagnosis. This allows the ISK technology to effectively capture the dispersed or drifting fault energy and to yield a single interpretable scalar with minimal parameterization, enabling reliable real-time monitoring after standard preprocessing (i.e., angular resampling, TSA and residual extraction).

Alongside vibration-based technologies, motor current signature analysis (MCSA) has emerged as a practical, non-intrusive alternative for gearbox monitoring. The mechanical faults, such as tooth cracks, eccentricities, and lack of lubrication, modulate the load torque, producing characteristic sidebands around the supply frequency and gear mesh harmonics in a motor current spectrum. An MCSA avoids the need for the direct mounting of vibration sensors and leverages the existing electrical infrastructure, making it attractive for industrial deployment.

The objective of this research is to propose, develop and experimentally validate a new fault diagnosis technology for gearboxes. Specifically, this study aims to evaluate the spectral kurtosis from residual vibration signals, apply the proposed novel integrated spectral kurtosis (ISK) technology, evaluate its total probability of correct diagnosis (TPCD), investigate the influence of the ISK threshold selection on the technology, and perform a systematic comparison of it with conventional SK-SE vibration diagnostic technology and motor current signature analysis (MCSA) technology, using identical experimental data.

The main novelties of this study are:

- An integrated spectral kurtosis technology that is conceptualized for fault diagnosis.
- The experimental diagnosis results related to the proposed technology.
- A comparison of the ISK technology and conventional SK technology.
- A comparison of the ISK technology and motor current signature analysis (MCSA) technology.

## 2. Theoretical Analysis

### 2.1. Vibration-Based Technologies

#### 2.1.1. Spectral Kurtosis Technology

The spectral kurtosis (SK) is a higher-order statistical tool used to detect non-Gaussian transients and impulsive components within vibration signals, features often associated with faults in gearboxes. Unlike classical kurtosis, which yields a single scalar value reflecting the impulsiveness of an entire signal, the SK estimates the kurtosis as a function of frequency, thereby localizing the non-stationary transients within a spectral domain [37].

Given a signal x(t), the SK is formally defined as [23]

(1) $$K_{x}(f)=\frac{E\left[{\left|X(f,t)\right|}^{4}\right]}{E{\left[{\left|X(f,t)\right|}^{2}\right]}^{2}}-2,$$

where X(f,t) is the time–frequency representation of x(t), typically estimated via a short-time Fourier transform (STFT) using a moving analysis window. The expectation $E\left[\cdot \right]$ denotes the averaging over time.

In practical implementations, x(t) is windowed into short overlapping segments $x_{n}$, and the SK is estimated as

(2) $$K_{x}(f)=\frac{\frac{1}{N}\sum_{n=1}^{N}{\left|X_{n}(f)\right|}^{4}}{{\left(\frac{1}{N}\sum_{n=1}^{N}{\left|X_{n}(f)\right|}^{2}\right)}^{2}}-2,$$

where N is the number of segments and $X_{n}\left(f\right)$ is the Fourier transform of the $n^{th}$ windowed segment.

The result is a spectral curve where the peaks indicate the frequency bands containing impulsive activities.

#### 2.1.2. Integrated Spectral Kurtosis (ISK) Technology

The integrated spectral kurtosis (ISK) is introduced as a global impulsiveness-based technology designed to quantify the non-Gaussian impulsive transients in signals. The technology integrates information across the entire frequency spectrum rather than restricting the analysis to selected bands. This technology enables diagnosis of dispersed or broadband impulsive activity that may not be confined to a narrow frequency region, thereby improving its effectiveness under varying operating conditions.

Let $K_{x}(f)$ denote the spectral kurtosis estimated at frequency bin f, and $S_{\alpha }$ be a statistical threshold. A binary selection function Γ(f) is defined to retain only those frequency components whose SK values exceed the threshold:

(3) $$\Gamma (f)=\left\{\begin{array}{l} 1\;\mathrm{for}\;K_{x}(f)>S_{\alpha }\\ 0\;\mathrm{otherwise} \end{array}\right.,$$

The integrated spectral kurtosis is then formulated as the thresholded summation over the full spectral domain:

(4) $$ISK=\sum_{f=f_{\min}}^{f_{\max}}K_{x}(f)\times \Gamma (f),$$

**Figure 1:**
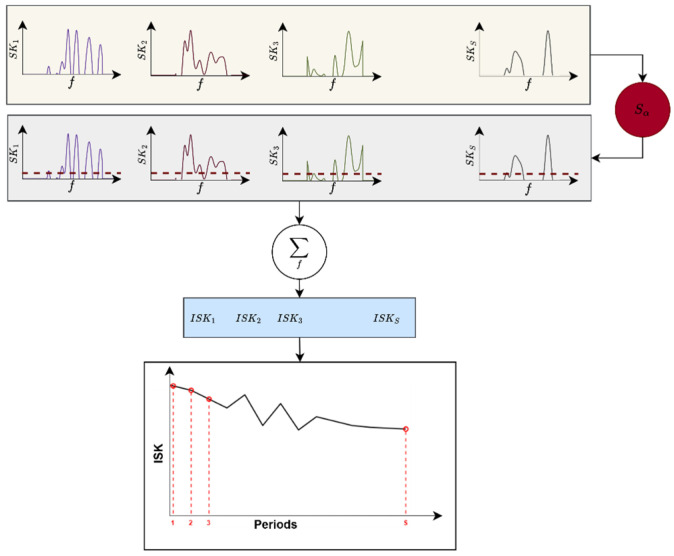
Flowchart of the proposed integrated spectral kurtosis technology.

This technology offers a compact measure of impulsive content across the entire spectrum, eliminating the need for frequency band selection and demodulation. Its global structure ensures the effective diagnosis of broadband transients and makes it well suited for real-time monitoring and fault diagnosis under non-stationary conditions. A flowchart of the proposed integrated spectral kurtosis technology is illustrated in Figure 1. The spectral kurtosis $K_{x}(f)$ is computed for each period (differentiated by colours), and a threshold $S_{\alpha }$ (horizontal dashed lines) is applied to retain the relevant components. These are then summed to obtain a single ISK value per period.

#### 2.1.3. Time Synchronous Averaging (TSA)

During the condition monitoring of rotating machinery, comparing the vibration signals across successive revolutions is essential for detecting fault-related patterns. However, because vibration data are typically acquired in the time domain under variable speed conditions, direct time-based comparisons can be unreliable. To address this, angular resampling is first applied to re-express the signals in the angular domain, enabling standardized comparisons across revolutions.

Let $s_{\mathrm{tach}}\left(t\right)$ represent a tachometer signal in the form of a rectangular waveform generated by an encoder that produces $n_{p}$ pulses per revolution. Let $x\left(t\right)$ be a time-domain vibration signal sampled at frequency $f_{s}$. The rising edges of $s_{\mathrm{tach}}\left(t\right)$ are detected at time indices $\left\{t_{1},t_{2},\ldots ,t_{M}\right\}$, where each $t_{i}$ corresponds to the $i^{th}$ detected encoder pulse. The total number of detected rising edges is denoted by M, which determines the maximum number of usable revolution segments.

Given that each revolution corresponds to $n_{p}$ pulses, the start and end of the $k^{th}$ full revolution occurs at time indices $t_{\left(k-1\right)n_{p}+1}$ and $t_{kn_{p}+1}$, respectively. The total number of complete revolutions that can be extracted from the tachometer signal is

(5) $$K=\left[\frac{M-1}{n_{p}}\right],$$

For each revolution $k\;\in \;\left\{1,\;2,\;\ldots ,\;K\right\},$ the segment of the signal corresponding to that rotation is

(6) $$x_{k}(t)=x(t),t\in \left[t_{(k-1)n_{p}},t_{kn_{p}+1}-1\right],$$

Since the number of time samples within each revolution varies with the speed, these segments must be resampled onto a fixed angular grid. To this end, a normalized angular vector is defined:

(7) $$\theta_{j}=\frac{j-1}{N-1},j=1,2,\ldots ,N,$$

where N is the desired number of angular samples per revolution. For each segment $x_{k}$, its original time base is similarly normalized:

(8) $$\tau_{i}^{k}=\frac{i-1}{L_{k}-1},i=1,2,\ldots ,L_{k},$$

with $L_{k}=t_{kn_{p}+1}-t_{\left(k-1\right)n_{p}+1}$ being the number of time-domain samples in revolution k.

The vibration signal is then resampled from its nonuniform domain $\tau^{\left(k\right)}$ to the uniform angular grid θ using interpolation. For each revolution k, the resampled angular signal is obtained as

(9) $${\tilde{x}}_{k}(\theta_{j})=\sum_{i=1}^{L_{k}}x_{k}(i).\phi (\theta_{j}-\tau_{i}^{k}),$$

where ϕ is the interpolation kernel.

The outcome is a set of uniformly sampled angular-domain signals ${\tilde{x}}_{k}\in R^{N}$, which can now be directly compared, averaged, or subjected to further processing, such as time synchronous averaging (TSA). Additionally, the angular sampling frequency becomes

(10) $$f_{\theta }=f_{s}\frac{N}{\bar{L}},$$

where $\bar{L}$ is the average number of time-domain samples per revolution.

Time synchronous averaging (TSA) is then applied to suppress the non-synchronous noise and emphasize the periodic components tied to shaft rotation, such as the gear mesh harmonics. By exploiting the angular alignment achieved during resampling, the TSA forms a composite signal by averaging R revolutions as [10,14]

(11) $$x_{TSA}(\theta )=\frac{1}{R}\sum_{k=1}^{R}{\tilde{x}}_{k}(\theta ),$$

This averaging operation retains the phase-consistent features while attenuating the stochastic and asynchronous disturbances [7,8]. The result is a smoothed representation of the signal over one typical revolution in the angular domain, ideal for revealing periodic mechanical behaviours. TSA has proven particularly effective for enhancing the fault-related components in gear transmission systems, such as mesh-induced vibrations, sidebands, and modulations [10,22,23].

#### 2.1.4. Classical Residual Signal

Despite the effectiveness of time synchronous averaging (TSA) for reinforcing the periodic components, the weak transients associated with gear faults may still be obscured by the dominant gear mesh signal. To enhance the detectability of such non-cyclic fault-induced events, the residual signal is estimated by subtracting a periodic approximation of the mesh component from the TSA waveform [10,21].

Let $x_{TSA}\left(\theta \right)$ denote the angular-domain TSA signal. If the meshing component corresponds to a gear with $N_{t}$ teeth, its angular period is given by $T_{m}=\frac{2\pi }{N_{t}}$. The periodic mesh component can then be estimated by averaging the shifted versions of the TSA signal:

(12) $$x_{mesh}(\theta )=\frac{1}{N_{t}}\sum_{n=0}^{N_{t}-1}x_{TSA}(\theta -nT_{m}),$$

The residual signal $r\left(\theta \right)$, which emphasizes the non-periodic, fault-related transients, is obtained as [11]

(13) $$r(\theta )=x_{TSA}(\theta )-x_{mesh}(\theta ),$$

**Figure 2:**
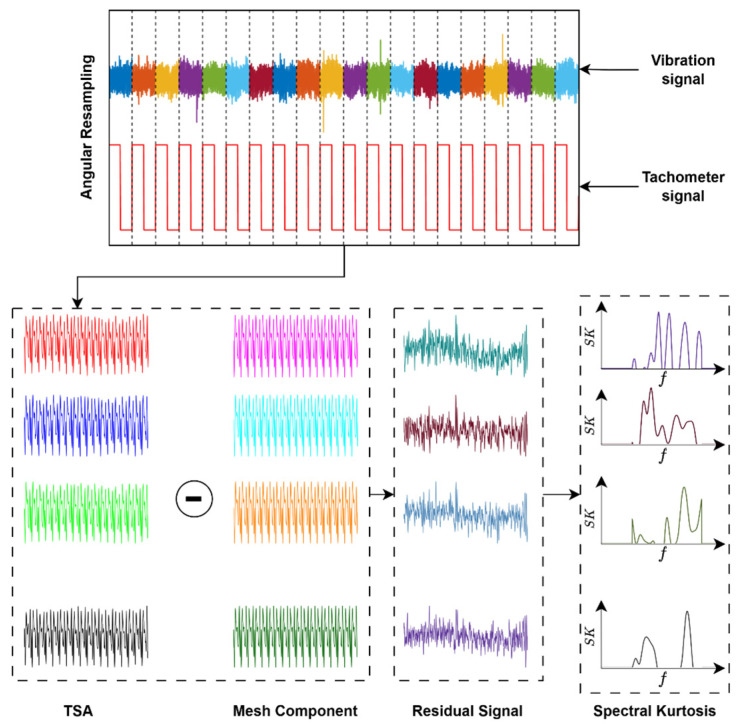
Illustration of different steps for estimation of time synchronous averaging (TSA), residual signal, and spectral kurtosis. Different colours represent successive signal segments (periods). The coloured plots show the time signals and their spectral kurtosis distributions for each segment. The red waveform denotes the pulse train used to define the segmentation.

This process effectively cancels the deterministic mesh content while preserving the low-energy impulsive components, thereby improving the effectiveness of subsequent fault diagnosis technologies, such as the spectral kurtosis (SK). The various steps for estimation of time synchronous averaging (TSA), residual signal, and spectral kurtosis are illustrated in Figure 2, where the vibration signal is first converted into the angular domain through angular resampling, then averaged over successive revolutions (shown in different colours, used to differentiate between periods) to obtain the TSA signal. The periodic mesh component is then estimated and subtracted from the TSA signal to obtain the residual signal, and finally the spectral kurtosis is estimated from the residual signal.

#### 2.1.5. Squared Envelope

Beyond fault localization, the spectral kurtosis (SK) facilitates the design of adaptive filters aimed at isolating transient events embedded within background noise [23]. The spectral kurtosis of the composite signal $y\left(t\right)=x\left(t\right)+n\left(t\right),$ where x(t) represents the non-stationary component and n(t) denotes the stationary Gaussian noise, is related to the spectral kurtosis of the non-stationary component x(t) through the following expression [23,33]

(14) $$K_{y}(f)=\frac{K_{x}(f)}{{\left[1+\rho (f)\right]}^{2}},$$

where $\rho \left(f\right)=\frac{S_{n}(f)}{S_{x}(f)}$ is the noise-to-signal ratio. $S_{x}(f)$ and $S_{n}\left(f\right)$ represent the power spectral densities of non-stationary component x(t) and stationary Gaussian noise $n\left(t\right),$ respectively.

The signal-to-noise ratio is high (i.e., f≈0) within the resonance bandwidth and low outside it. Consequently, $K_{y}(f)\approx K_{x}(f)$ within this bandwidth, while $K_{y}(f)\approx 0$ elsewhere [33]. Under these conditions, the Wiener filter becomes proportional to the square root of the spectral kurtosis, allowing the SK to determine the optimal filter $W\left(f\right)\;$ for isolating the transient components from the stationary background noise [33].

(15) $$W(f)=\left\{\begin{array}{l} \sqrt{K_{x}(f)}\;\mathrm{for}\;K_{x}(f)>S_{\alpha }\\ 0\;\mathrm{otherwise}\; \end{array}\right.$$

where $K_{x}(f)$ denotes the spectral kurtosis estimate and $S_{\alpha }$ is a statistical threshold defining the separation between the impulsive and noise-dominated frequency regions [9,38].

The SK-residual signal in the frequency domain is then constructed as

(16) $$R_{SK}(f)=W(f)\times X(f)$$

where X(f) is the Fourier transform of the original vibration signal x(t). Applying the inverse Fourier transform yields the SK-residual signal $r_{SK}$, which contains the enhanced transient content:

(17) $$r_{sk}(t)=iFFT\left(R_{sk}(f)\right)$$

To further amplify the impulsive modulations, the envelope of $r_{SK}$ is estimated using a Hilbert transform H{⋅}, yielding a demodulated signal that highlights the non-stationary amplitude variations [19]. The final diagnostic feature, known as the squared envelope, is defined as

(18) $$SE(t)={\left|r_{sk}(t)+j.H\left\{r_{sk}(t)\right\}\right|}^{2},$$

### 2.2. Motor Current-Based Technology

Gear faults, such as pitting, cracking, and wear, are well known to introduce modulation effects in gearbox vibration signals [39]. When a damaged tooth engages in meshing, it produces abrupt disturbances that manifest as both amplitude modulation (AM) and frequency modulation (FM) in the vibration response. These modulations occur periodically at the characteristic frequency of the defected gear, giving rise to sidebands around the gear mesh frequency and its harmonics in the spectral domain [39]. Such AM-FM features have been consistently reported as reliable fault indicators, distinguishing gear faults from normal manufacturing or load-related errors [39]. Therefore, the mesh harmonics and their sidebands can be considered key spectral features for gearbox fault diagnosis. Complementing this vibration analysis, the gear mesh-induced torque oscillations modulate the induction motor stator current under gear faults: the current’s dominant frequency exhibits both amplitude and phase modulation, producing fault-related sidebands that evolve over time around the power supply frequency and its harmonics. These AM-FM gear mesh signatures and their sideband distributions for gearbox faults have been analytically derived and experimentally validated for non-stationary conditions, supporting the use of motor current as a practical diagnostic technology [16].

In gear-driven systems, the fundamental mesh frequency $f_{mesh}$ is a key diagnostic feature and is defined as

(19) $$f_{mesh}=N.f_{r},$$

where N is the number of teeth on a gear and $f_{r}$ is the rotational frequency of the corresponding shaft.

In multi-stage gearboxes, each gear pair generates its own mesh frequency, determined by the gear ratio and geometry. These mesh frequencies and their harmonics vary with the load and operating conditions, and their amplitudes are linked to the dynamic behaviour of the gearbox.

Theoretical models of gearbox dynamics show that faults, such as pitting, cracks, or scuffing, introduce amplitude modulations at these mesh frequencies. The spectral representation of a monitored signal therefore contains the harmonics of $f_{mesh},$ whose amplitudes reflect the energy associated with gear meshing. The unnormalized mesh harmonic amplitude (UMHA) is defined as the raw spectral amplitude at the mesh frequency or its harmonics and provides a direct measure of energy concentration.

## 3. Methodology

The time-domain vibration signals are first converted into the angular domain using the tachometer pulses, ensuring uniform angular sampling and the consistent alignment of periodic components across revolutions. The tachometer signal consists of a pulse train with one pulse per shaft revolution, which is used as the angular reference for resampling. Time synchronous averaging (TSA) is then applied to enhance the deterministic gear mesh harmonics and suppress the non-synchronous noise. The TSA is estimated over 80 consecutive revolutions. The classical residual signal (CRS) is subsequently obtained by subtracting the periodic mesh component from the TSA waveform, isolating the non-periodic impulsive transients linked to gear faults. The CRS is particularly suitable for impulsive fault diagnosis, as it suppresses the deterministic meshing components while retaining the non-periodic, impact-related transients.

The spectral kurtosis (SK) is estimated from the residual signals to identify the frequency bands exhibiting high impulsiveness. The spectral kurtosis estimation is performed using a short-time Fourier transform with a Hamming window; a window length is equal to half the gear mesh period, with an overlap ratio of 80%. These SK values are used to design adaptive filters that emphasize the transient-dominant regions. The filtered signals are analytically represented via a Hilbert transform, and the squared envelope (SE) is derived from the instantaneous amplitude to quantify the energy associated with the fault-induced impacts.

While the conventional SK-based technology relies on the frequency-band selection, adaptive filtering, and envelope extraction, the proposed integrated spectral kurtosis (ISK) technology is obtained directly from the SK spectrum without an additional filtering or an envelope analysis. By summing all the SK values exceeding a threshold across the entire frequency range, the ISK provides a single value that captures the total impulsive energy content. To further enhance the total probability of correct diagnosis (TPCD), a band similarity analysis is performed to identify the frequency regions exhibiting consistently high SK values in both healthy and defective conditions. The SK values from multiple realizations are organized into a “frequency-realization” matrix, and the mean SK spectrum is estimated to identify the dominant impulsive regions. The candidate frequency bands are defined around bell-shaped SK peaks exceeding a predefined threshold, with the band boundaries determined by adjacent threshold crossings or local minima. The band persistence is quantified by detecting the local SK peaks in each realization using adaptive percentile-based thresholds and estimating the percentage of realizations in which at least one peak occurs within each band. In parallel, a peak consistency-based detection methodology is used to estimate the frequency range probability by tracking persistent SK peaks across realizations, and a band confidence metric is then estimated as the percentage of realizations activating each band. The bands exhibiting high confidence in both healthy and defected conditions are excluded from the ISK estimation.

The threshold for the ISK technology has an optimum value (or optimum threshold range) at low thresholds, which result in a TPCD ≥90%, whereas large thresholds reduce the TPCD to 50%, which confirms the existence of an optimum threshold (or optimum threshold range). For this study, the optimum threshold is T=0 for pinion 1 and T [0.03] for gear 1. To determine the optimum threshold range, the methodology proposed here is based on a TPCD–threshold dependency, in which the threshold is varied from 0 up to a maximum value, the ISK is estimated at each threshold, and an optimum threshold range is defined as value(s) of threshold for which the TPCDs are maximal. A threshold range from 0 to 0.9 is used in this study to identify the optimum threshold range.

The motor current-based diagnostic technology is designed to exploit the electromechanical coupling between the drive motor and gearbox. The stator current signal is first divided into consecutive segments to facilitate time–frequency tracking. Each segment is then converted into its analytic form, and the envelope is extracted to emphasize the amplitude modulations associated with the periodic torque fluctuations induced by gear meshing and potential defects.

**Figure 3:**
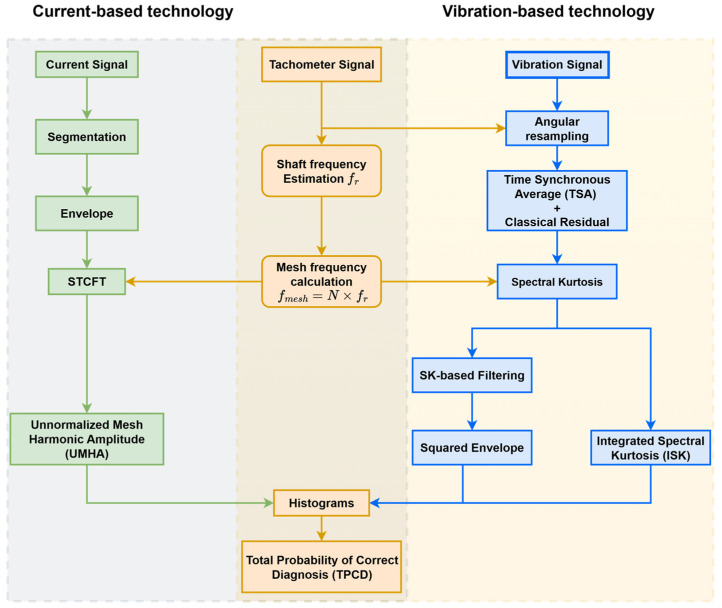
Schematic of vibration-based and current-based methodologies for diagnostic feature extraction.

The envelope signal is subsequently analysed using a short-time chirp Fourier transform (STCFT) [38,40]. From the STCFT spectrum, the diagnostic features are extracted based on the spectral magnitude [41] of the gear mesh harmonics. The unnormalized mesh harmonic magnitude (UMHA) represents the raw spectral peak magnitude at the instantaneous mesh frequency. The instantaneous gear mesh frequency is estimated using two dependencies: the tachometer signal and the STCFT spectrum. First, the gear mesh frequency is estimated from the tachometer signal. This estimated frequency is then used to track and localize the corresponding mesh harmonics in the STCFT frequency axis. Since the estimated mesh frequency does not always coincide with an exact discrete frequency bin in the STCFT spectrum, a local bin selection strategy is adopted. For each time segment, the STCFT bin closest to the estimated mesh frequency, together with one adjacent bin on either side, is considered. The highest magnitude among these three bins is then selected to represent the harmonic amplitude. The complete methodology integrating both the vibration-based and current-based diagnostic technologies is illustrated in Figure 3.

To evaluate its diagnostic effectiveness, the Gaussian probability density functions (PDFs) are fitted to the feature distributions. For a normally distributed random variable x with mean μ and standard deviation σ, the PDF is defined as [42]

(20) $$PDF(x)=\frac{1}{\sqrt{2\pi \sigma^{2}}}\exp\left(-\frac{{\left(x-\mu \right)}^{2}}{2\sigma^{2}}\right),$$

The classification threshold is defined as the Bayesian intersection [43] of the two PDFs, assuming the mean of the defective class exceeds that of the healthy class. The total probability of correct diagnosis (TPCD) is then estimated as

(21) $$TPCD=\frac{1}{2}\frac{N_{D}+N_{H}}{N_{Dt}+N_{Ht}}$$

where $N_{D}$ and $N_{H}$ are the numbers of correctly classified defected and healthy cases, respectively, and $N_{Dt}$ and $N_{Ht}$ denote the total number of assessed defected and healthy cases.

The fault diagnosis methodology implemented in this study consists of signal acquisition, angular resampling using tachometer pulses, time synchronous averaging, residual signal extraction, diagnostic features estimation (the ISK, SK-SE and UMHA), band similarity analysis and the TPCD estimation.

## 4. Experimental Setup

**Figure 4:**
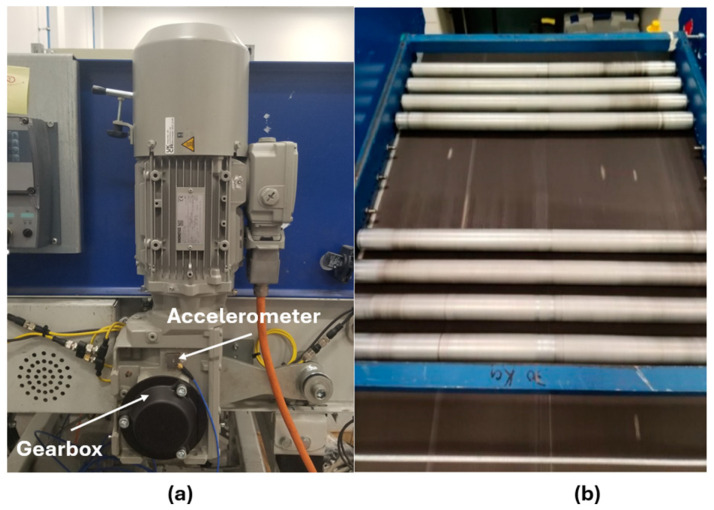
The experimental setup: (**a**) the gearmotor of the conveyor belt system and (**b**) the loaded conveyor belt system [42].

The test rig replicates an airport baggage handling conveyor system and is driven by a Siemens JKE2104 gearmotor (Siemens, Munich, Germany), integrating a three-phase induction motor with a two-stage gearbox. The motor operates at a nominal supply frequency of 50 Hz and a rated speed of 1440 rpm. A 20 kg additional load is applied using rollers mounted in a rigid frame placed on the conveyor belt (Figure 4).

The vibration measurements are performed with a PCB Piezotronics 354A05 triaxial ICP^®^ accelerometer (PCB Piezotronics, Depew, NY, USA) mounted on the gearbox housing. The sensor employs a ceramic shear element in a titanium casing, includes TEDS (IEEE 1451.4), and provides 100 mV/g sensitivity, ±50 g dynamic range, and a flat frequency response from 0.4 Hz to 5 kHz (±5%). It is powered by a constant current source (2–20 mA). Three LEM AT-B10 split-core current transducers (LEM, Geneva, Switzerland) are used to monitor the motor current, with one per phase. These non-intrusive sensors provide galvanic isolation and a 0–10 V DC output proportional to the RMS current, with ±0.5% linearity and 50/60 Hz operating range. Signal conditioning is performed using KEMO DR-series anti-aliasing filters (KEMO Ltd., Thetford, UK). A DR1200 is employed for the vibration signals, providing a 4 mA current source for the IEPE sensors and a 20 kHz low-pass cutoff. A DR1600 is used for the motor current signals, configured with a 5.1 kHz cutoff and ×20 gain. Both units provide <0.003% THD and gain settings up to ×1000 in 1-2-5 steps.

**Figure 5:**
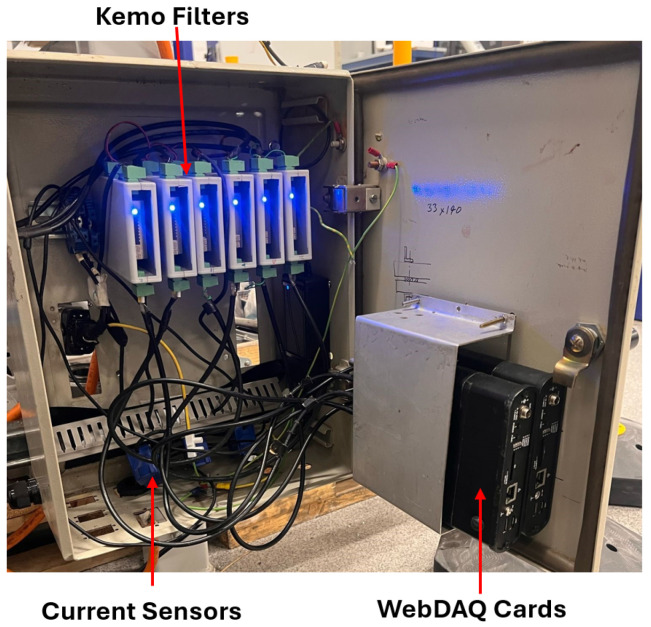
Data acquisition system.

**Figure 6:**
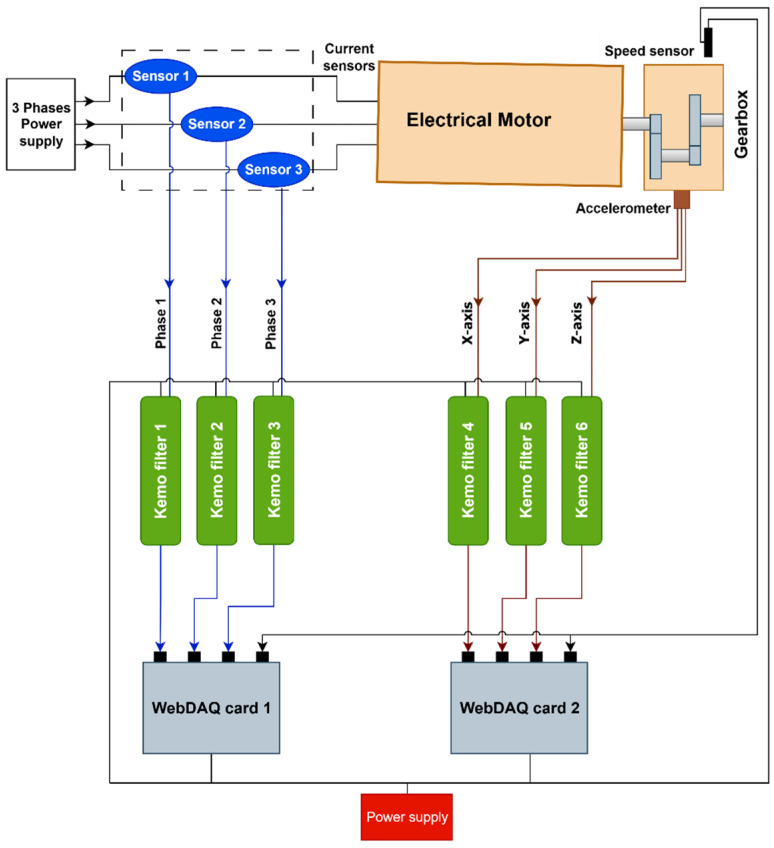
Schematic of the data acquisition system.

The shaft rotational speed is tracked with a VLS5-D-LSR laser tachometer (Compact Instruments Ltd., Bolton, UK), using reflective tape on the gearbox output shaft. The sensor operates from 3 to 250,000 rpm, with a working distance of 50 mm–2 m and a ±80° angular tolerance. All signals are acquired using a WebDAQ 504 system (24-bit resolution, ±5 V range, 51.2 kHz maximum sampling rate per channel). The unit allows for the synchronized acquisition of vibration and tachometer signals with real-time monitoring (Figure 5 and Figure 6).

The gearbox investigated in this study is a two-stage transmission comprising a helical and a bevel stage. The first stage is a helical gear pair consisting of an 18-tooth pinion meshing with a 37-tooth gear at a 30° helix angle, giving a transmission ratio of 18/37. The second stage is a bevel gear pair formed by a 17-tooth pinion and a 46-tooth gear, corresponding to a ratio of 17/46. For clarity, the following nomenclature is adopted: pinion 1 denotes the helical pinion on the input shaft, gear 1 refers to the helical gear on the intermediate shaft, pinion 2 designates the bevel pinion on the intermediate shaft, and gear 2 corresponds to the bevel gear on the output shaft.

Two operating conditions were assessed under a constant applied load of 20 kg. In the healthy condition, the gearbox operated with clean oil filled to the manufacturer’s recommended level, representing the nominal maintenance state. In the defected condition, the post-test inspection revealed that the gearbox had run with contaminated oil, which had degraded during operation. The contamination introduced aluminium debris into the lubrication system, altering the film formation and promoting asperity contact between the meshing teeth.

**Figure 7:**
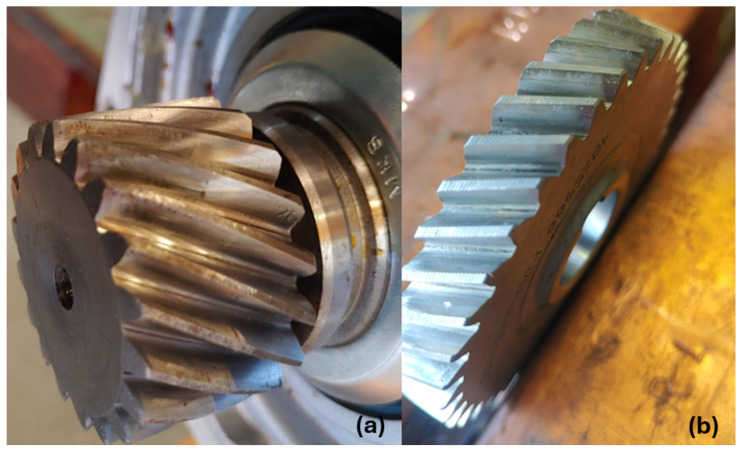
The inspected gearbox wheels: (**a**) helical pinion and (**b**) helical gear.

The inspection findings confirmed that the effects of oil contamination were most pronounced in the helical stage. On pinion 1 (Figure 7a), the drive flank displayed a mixture of early-stage micro-pitting and light scoring. The contact pattern covered the full flank width, yet it exhibited a distinct “bow-shaped” distortion, which can be attributed to the excessive end float measured on the first wheel line. This misalignment, combined with the abrasive effect of aluminium particles, explains the observed scoring and the initiation of pitting. On gear 1 (Figure 7b), the driven flank also showed early micro-pitting, with the score marks concentrated near the tooth tip, again consistent with contamination-driven abrasion.

By contrast, the bevel stage remained unaffected. Both pinion 2 and gear 2 exhibited polished drive and driven flanks characteristic of the bedding-in process of hardened and lapped gears. At this stage, no evidence of pitting was detected.

In summary, the investigation shows that oil contamination strongly accelerates surface faults in the helical stage, with micro-pitting and scoring already visible on pinion 1 and gear 1. The bevel stage remains healthy.

## 5. Diagnosis Results and Discussion

### 5.1. Vibration Technology Results

The vibration and rotational speed data are acquired every 20 min, with each recording lasting 1200 s. To enable a rotation-consistent analysis, the vibration signals are converted from the time to the angular domain using reference pulses from the tachometer. Since the tachometer is mounted on the output shaft of a two-stage gearbox with three rotating shafts (input, intermediate, and output), the tachometer signals for the upstream shafts are reconstructed from the known gear ratios. The shaft rotational speeds are estimated using the elapsed-time method, which estimates the speed from the time intervals between successive tachometer pulses [44].

The angular-domain vibration signals are processed using time synchronous averaging (TSA) to extract the periodic components associated with gear meshing. The number of averaged rotations is determined based on the energy convergence criteria [13], ensuring stability of the averaged waveform while enhancing visibility of gear-induced modulation patterns and suppressing non-synchronous disturbances. Based on this criterion, 80 revolutions are selected for averaging.

The spectral kurtosis (SK) is estimated for the residual signals of each gear stage under both healthy and defected conditions. The SK estimation employs a short-time Fourier transform (STFT), with each residual signal segmented by a Hamming window of length $N_{w}$. Following previous studies [37,45], the window length is set to half the angular mesh period of the gear under analysis to balance the frequency resolution and transient capture within a single meshing cycle. An overlap of 80% is applied between successive windows to improve the temporal continuity and reduce the spectral leakage.

**Figure 8:**
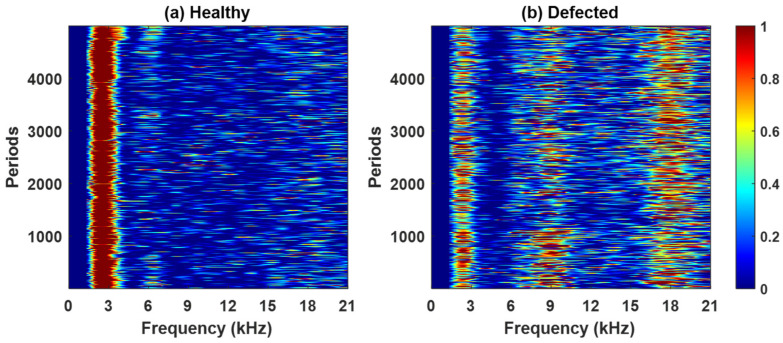
The spectral kurtosis for pinion 1 residual signal: (**a**) healthy, (**b**) defected.

**Figure A1:**
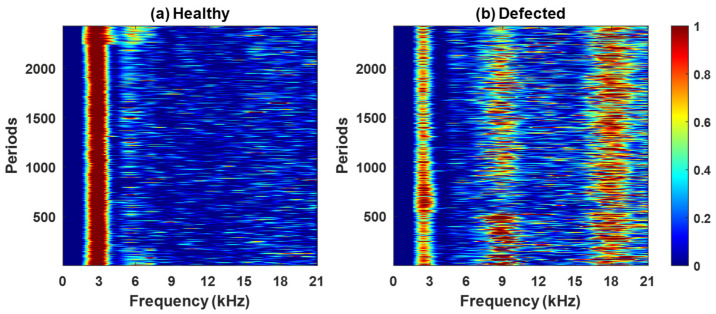
The spectral kurtosis for gear 1’s residual signal: (**a**) healthy, (**b**) Defected.

**Figure A2:**
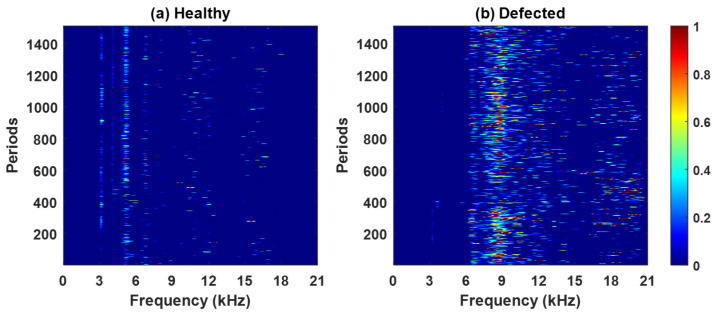
The spectral kurtosis for pinion 2’s residual signal: (**a**) healthy, (**b**) defected.

**Figure A3:**
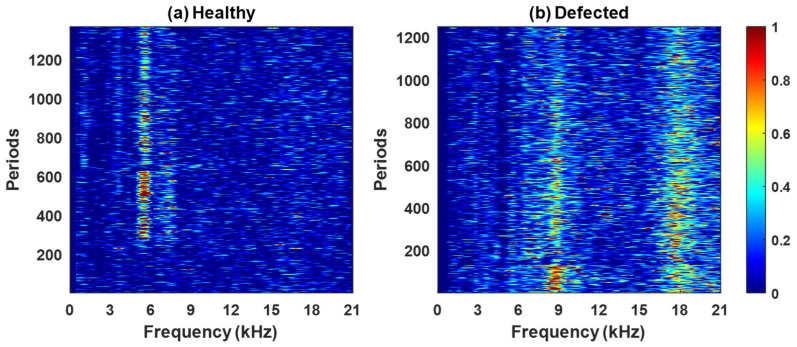
The spectral kurtosis for gear 2’s residual signal: (**a**) healthy, (**b**) defected.

Figure 8, Figure A1, Figure A2 and Figure A3 (Appendix A) present the spectral kurtosis (SK) maps for pinion 1, gear 1, pinion 2, and gear 2 under healthy and defected conditions. These period-frequency representations illustrate the evolution of impulsive content across successive gear rotations. In the healthy case (Figure 8a, Figure A1a, Figure A2a and Figure A3a), the SK values remain low and broadly distributed, consistent with normal meshing and the absence of strong impulsive events. For pinion 1 (Figure 8a) and gear 1 (Figure A1a), elevated responses occur near 3 kHz and 6 kHz but decrease under the defected conditions, suggesting an association with structural resonances. For pinion 2 (Figure A2a), peaks appear near 3 kHz and 5 kHz. Gear 2 (Figure A3a) exhibits moderate SK activity around 6-7 kHz, reflecting low-level impulsiveness not attributable to a fault. In contrast, the defected case (Figure 8b, Figure A1b, Figure A2b and Figure A3b) shows markedly higher SK responses, particularly within the 9 kHz and 18 kHz bands. These peaks are sharper, more energetic, and persist across multiple rotations, indicating repeated impulsive events typical of localized gear fault. Pinion 1 and gear 1 exhibit the most pronounced activity, whereas pinion 2 and gear 2 present lower amplitudes but still concentrate in the same frequency bands.

**Figure 9:**
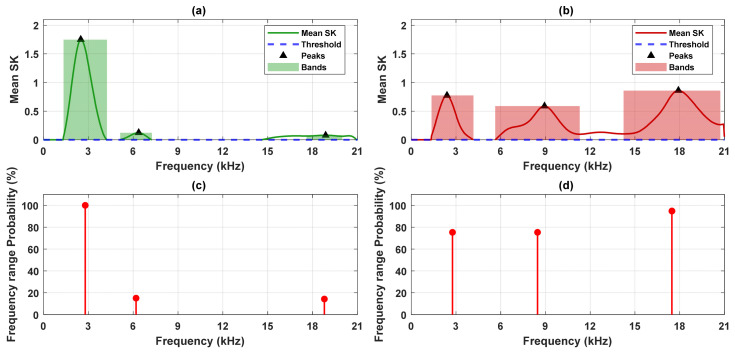
Frequency-wise probability of persistent SK peaks and corresponding band confidence levels used for band similarity analysis between healthy and defected conditions for pinion 1. (**a**) The mean spectral kurtosis (healthy), (**b**) mean spectral kurtosis (defected), (**c**) frequency range probability (healthy), and (**d**) frequency range probability (defected). The red shaded regions represent the frequency bands identified under defected conditions, while the green shaded region indicates the corresponding band under healthy conditions.

Establishing the spectral distribution of impulsiveness under healthy gearbox conditions is essential for reliable diagnostics. This baseline allows for future measurements to be compared, where deviations or the emergence of new peaks in the spectral kurtosis (SK) can be interpreted as fault indicators. Figure 9 illustrates this approach, presenting the mean SK spectra for both healthy and defected cases, averaged over all gear rotation periods. Averaging highlights the consistently active frequency bands and supports the diagnosis of normal behaviour and fault-induced transients.

**Figure A4:**
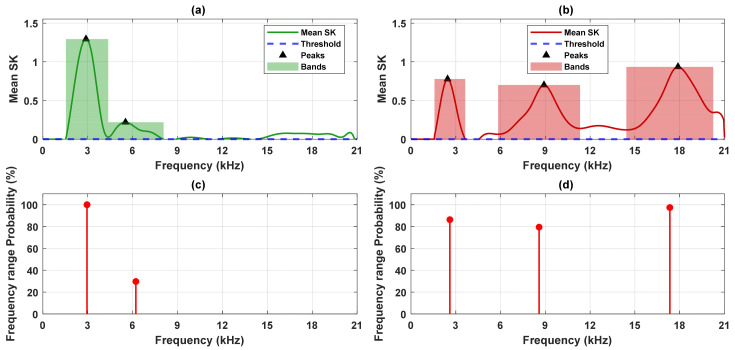
Frequency-wise probability of persistent SK peaks and corresponding band confidence levels used for band similarity analysis between healthy and defected conditions for gear 1. (**a**) The mean spectral kurtosis (healthy), (**b**) mean spectral kurtosis (defected), (**c**) frequency range probability (healthy), and (**d**) frequency range probability (defected). The red shaded regions represent the frequency bands identified under defected conditions, while the green shaded region indicates the corresponding band under healthy conditions.

**Figure A5:**
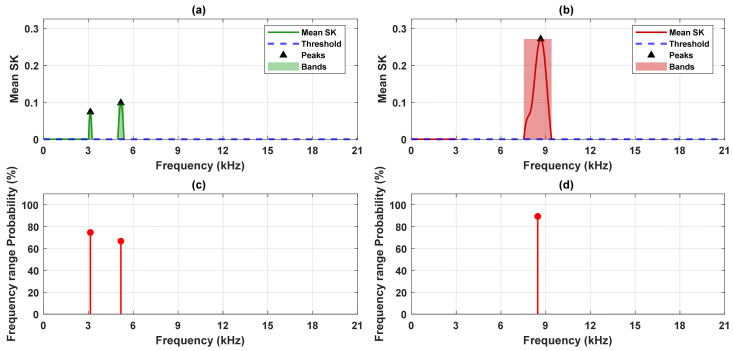
Frequency-wise probability of persistent SK peaks and corresponding band confidence levels used for band similarity analysis between healthy and defected conditions for pinion 2. (**a**) The mean spectral kurtosis (healthy), (**b**) mean spectral kurtosis (defected), (**c**) frequency range probability (healthy), and (**d**) frequency range probability (defected). The red shaded regions represent the frequency bands identified under defected conditions, while the green shaded region indicates the corresponding band under healthy conditions.

**Figure A6:**
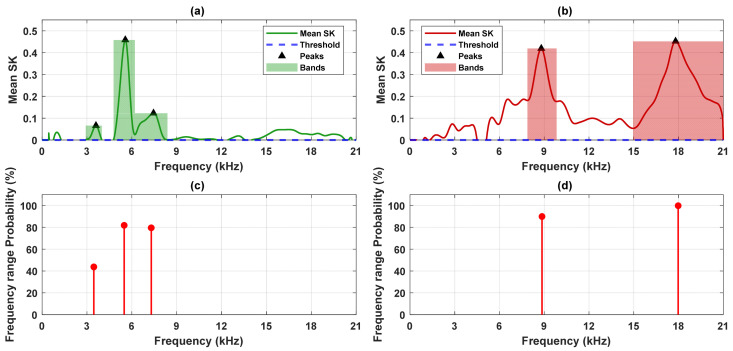
Frequency-wise probability of persistent SK peaks and corresponding band confidence levels used for band similarity analysis between healthy and defected conditions for gear 2. (**a**) The mean spectral kurtosis (healthy), (**b**) mean spectral kurtosis (defected), (**c**) frequency range probability (healthy), and (**d**) frequency range probability (defected). The red shaded regions represent the frequency bands identified under defected conditions, while the green shaded region indicates the corresponding band under healthy conditions.

For the healthy case (Figure 9a), the SK spectrum shows a dominant peak at 3 kHz and a smaller peak near 6 kHz. These bands are linked to structural resonances. No significant SK activity is observed beyond 6 kHz, indicating a stable profile at higher frequencies. By contrast, the defected case (Figure 9b) demonstrates a markedly different distribution: while the 3 kHz component persists at a lower magnitude, two additional peaks appear at 9 kHz and 18 kHz. These new high-frequency bands are absent in the healthy condition and are attributed to impulsive transients induced by gear fault. Figure 9c,d present the frequency range probability estimated from the SK maps to quantify the consistency of impulsive features across gear rotations. The frequency range probability indicates the percentage of realizations with a peak within a predefined band. In the healthy condition (Figure 9c), the 3 kHz peak shows 100%, confirming its persistent presence and its role as a baseline characteristic. A smaller 6 kHz peak appears with 15% confidence, suggesting sporadic activity not representative of consistent impulsiveness. Under defected conditions (Figure 9d), strong and consistent peaks emerge at 9 kHz (75%) and 18 kHz (95%). The 3 kHz and 6 kHz bands remain, but with reduced intensity and lower probability (75%), reinforcing their interpretation as a structural resonance rather than a fault feature. The corresponding results for gear 1, pinion 2, and gear 2 are presented in Figure A4, Figure A5 and Figure A6.

**Figure 10:**
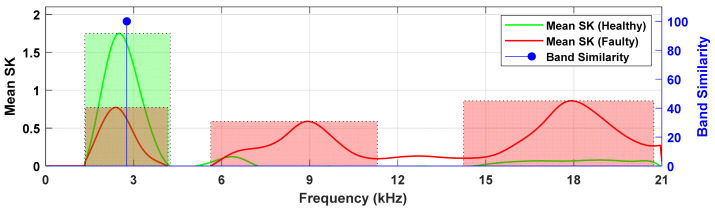
Band similarity analysis for pinion 1, comparing frequency bands detected in healthy and defected conditions. The red shaded regions represent the frequency bands identified under defected conditions, while the green shaded region indicates the corresponding band under healthy conditions.

**Figure A7:**
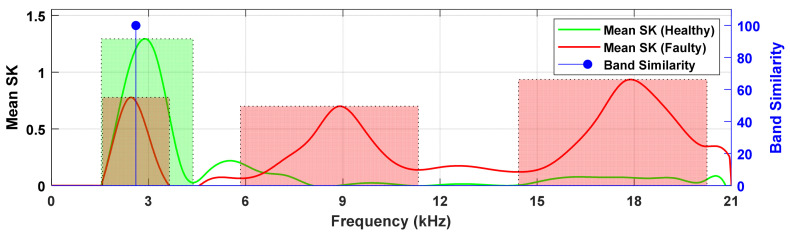
Band similarity analysis for gear 1, comparing frequency bands detected in healthy and defected conditions. The red shaded regions represent the frequency bands identified under defected conditions, while the green shaded region indicates the corresponding band under healthy conditions.

**Figure A8:**
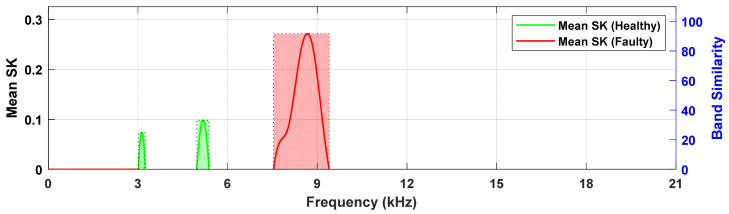
Band similarity analysis for pinion 2, comparing frequency bands detected in healthy and defected conditions. The red shaded regions represent the frequency bands identified under defected conditions, while the green shaded region indicates the corresponding band under healthy conditions.

**Figure A9:**
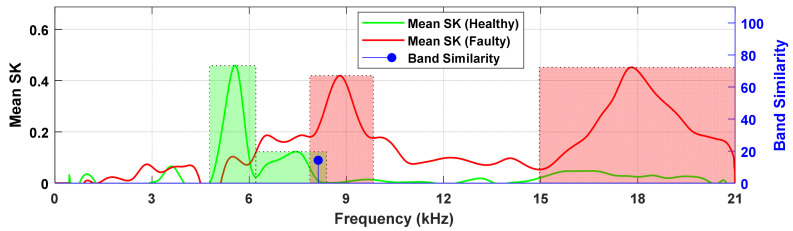
Band similarity analysis for gear 2, comparing frequency bands detected in healthy and defected conditions. The red shaded regions represent the frequency bands identified under defected conditions, while the green shaded region indicates the corresponding band under healthy conditions.

A band similarity assessment is performed to compare the spectral content of healthy and defected signals. The objective is to identify and exclude the frequency bands associated with inherent structural resonances rather than gear faults. As shown in Figure 10 and Figure A7, the 3 kHz band, observed in both healthy and defected cases, exhibits similarity, and is, therefore, classified as a non-diagnostic structural resonance. In contrast, the association of the 9 kHz and 18 kHz bands with the impulsive transients generated by gear fault is confirmed. In contrast to pinion 1 and gear 1, the band similarity analysis for pinion 2 and gear 2 (Figure A8 and Figure A9) do not reveal any overlapping frequency bands between the healthy and defected states.

**Figure 11:**
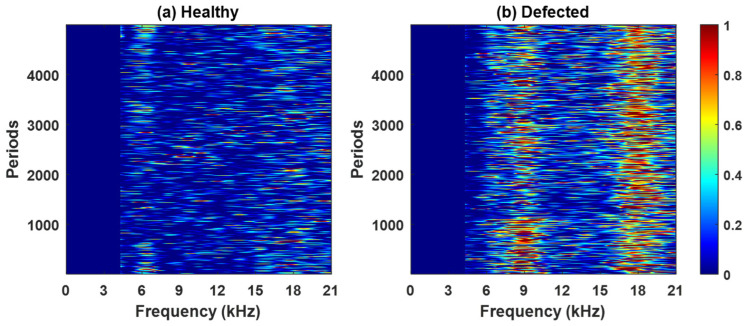
The updated SK colormaps for pinion 1 after excluding the 3 kHz band: (**a**) healthy, (**b**) defected.

**Figure A10:**
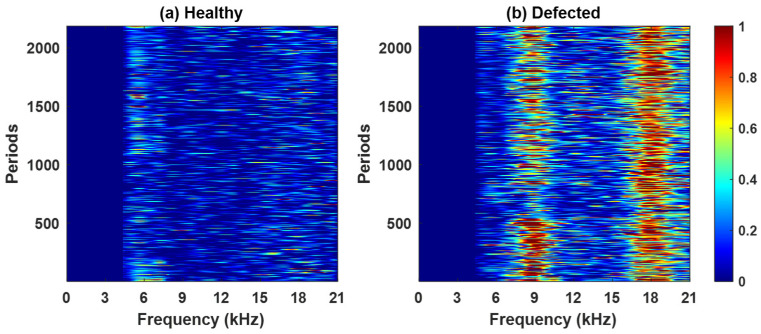
The updated SK colormaps for gear 1 after excluding the 3 kHz band: (**a**) healthy, (**b**) Defected.

Based on the band assessment, the 3 kHz band, which is very intensive in healthy conditions, is excluded from further analysis. To assess the impact of this exclusion, the diagnostic features, the squared envelope (SE) and the integrated spectral kurtosis (ISK), are evaluated before (version 1) and after (version 2) the band’s exclusion. The updated SK colormaps for pinion 1 are shown in Figure 11 (for gear 1, see Figure A10).

Following estimation of the spectral kurtosis (SK), two diagnostic technologies are utilized across the gear stages. The first is the conventional squared envelope (SE) technology, obtained via the SK-based band selection, narrowband filtering, and the Hilbert transform demodulation. This technology highlights the impulsive activity by estimating the instantaneous energy of the envelope signal and has been widely used for condition monitoring of rotating machinery. The second is the proposed integrated spectral kurtosis (ISK) technology, defined as a scalar value obtained by summing the thresholded SK values across all frequency bins. Unlike SE, ISK does not require band selection or demodulation. Instead, it quantifies the total impulsiveness of the residual signal. The following subsections present and compare the SE and the ISK technologies for each gear stage.

Based on the estimated SK, a threshold $S_{\alpha }$ is applied to retain the frequency bins with $K_{x}(f)>S_{\alpha }$, ensuring preservation of non-Gaussian components associated with a fault. A Wiener filter is then constructed in the frequency domain, with the filter gains proportional to the square root of the SK values. The SK-filtered residual signal is subsequently transformed into analytic form using a Hilbert transform, and the squared magnitude of the envelope is estimated. The resulting SE provides a time-tooth domain representation of instantaneous power fluctuations.

**Figure 12:**
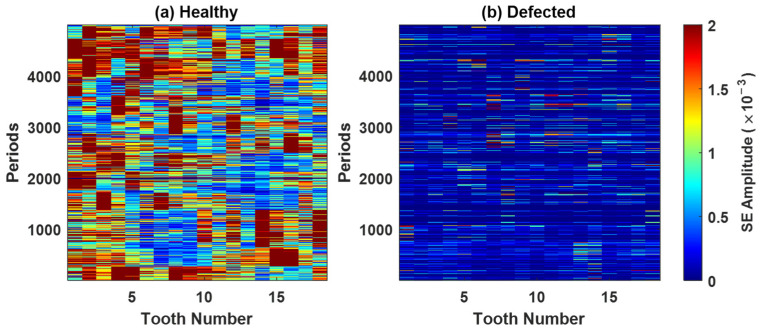
The squared envelope of the SK-filtered residual signal for pinion 1: (**a**) healthy, (**b**) defected.

**Figure 13:**
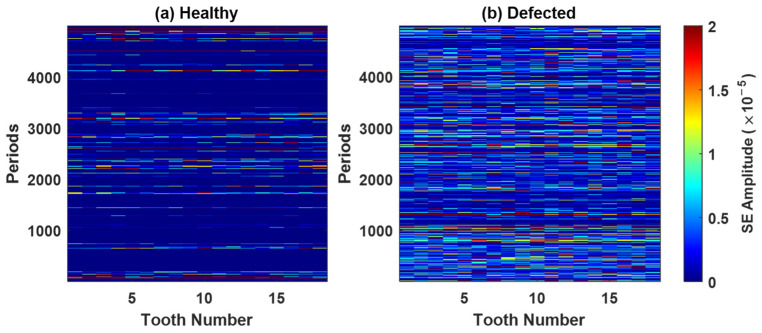
The squared envelope of the SK-filtered residual signal for pinion 1 after excluding the 3 kHz band: (**a**) healthy, (**b**) defected.

**Figure A11:**
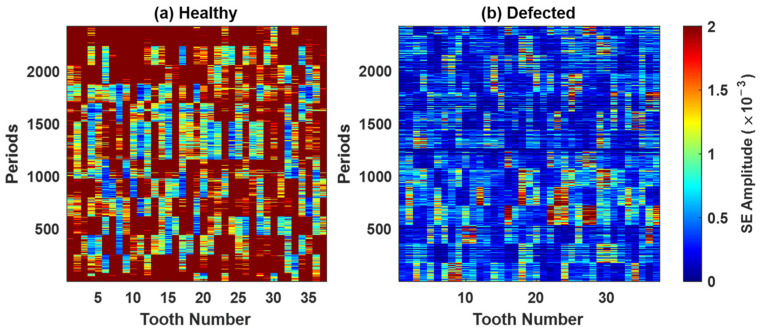
The squared envelope of the SK-filtered residual signal for gear 1: (**a**) healthy, (**b**) defected.

**Figure A12:**
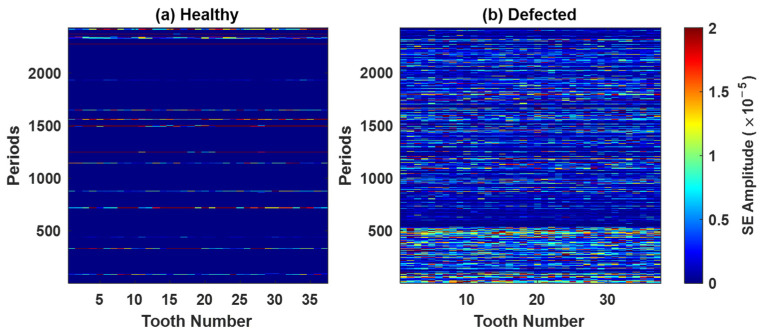
The squared envelope of the SK-filtered residual signal for gear 1 after excluding the 3 kHz band: (**a**) healthy, (**b**) defected.

**Figure A13:**
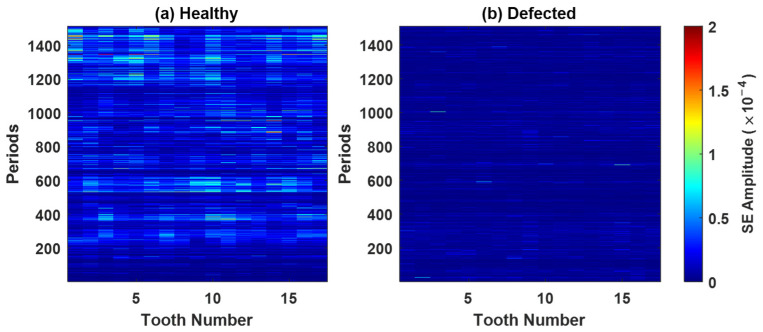
The squared envelope of the SK-filtered residual signal for pinion 2: (**a**) healthy, (**b**) defected.

**Figure A14:**
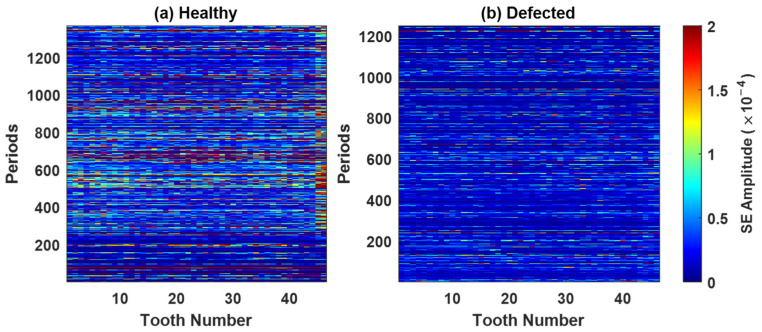
The squared envelope of the SK-filtered residual signal for gear 2: (**a**) healthy, (**b**) defected.

Figure 12 shows the SE maps of pinion 1 under (a) healthy and (b) defected conditions, using the full SK frequency range without band exclusion. In the healthy case, the SE amplitudes are higher and more broadly distributed across the teeth and periods, primarily due to the persistent contributions from the 3 kHz band. In the defected case, the SE amplitudes are lower overall but more localized, reflecting the transient impulses from a gear fault. The 3 kHz band, previously identified, is excluded. The updated SE maps are presented in Figure 13. In the healthy case (Figure 13a), the SE amplitudes are reduced, confirming the non-fault origin of the earlier high energy. In contrast, the defected case (Figure 13b) retains distinct impulsive signatures localized in both time and tooth position. The corresponding results for gear 1, pinion 2, and gear 2, before and after band exclusion, are provided in Figure A11, Figure A12, Figure A13 and Figure A14.

Based on the results in Figure 12 and Figure 13, histograms of the squared envelope of the SK-filtered residual signals are constructed and used to estimate the total probability of correct diagnostics (TPCD) for each tooth across all gear stages.

**Figure 14:**
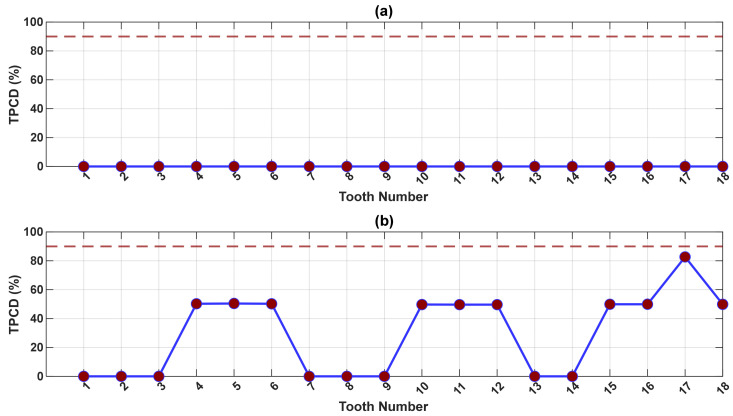
The total probability of correct diagnostics (TPCD) for the SK technology for pinion 1, evaluated across all teeth (**a**) before and (**b**) after excluding the 3 kHz band. The red dashed line indicates the 90% TPCD threshold; values above this threshold correspond to effective diagnosis. Coloured dots show TPCD values (red: non-effective).

**Figure A15:**
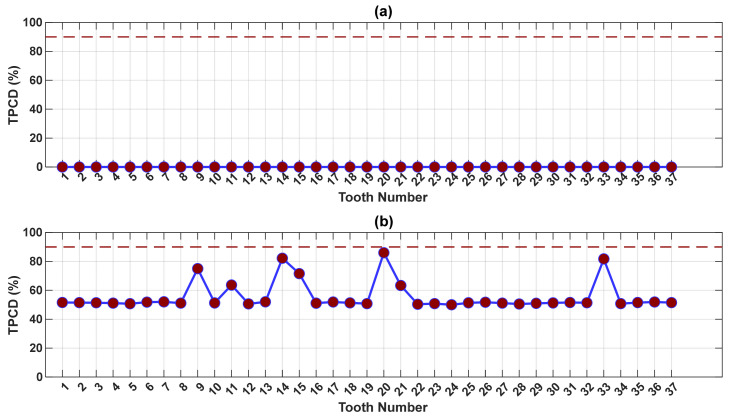
The total probability of correct diagnostics (TPCD) for the squared envelope of the SK-filtered residual signal for gear 1, evaluated across all teeth before and after excluding the 3 kHz band: (**a**) before, (**b**) after. The red dashed line indicates the 90% TPCD threshold; values above this threshold correspond to effective diagnosis. Coloured dots show TPCD values (red: non-effective).

As shown in Figure 14, for pinion 1, the TPCD before exclusion of the 3 kHz band (Figure 14a) remains uniformly poor across all teeth, with values equal to 0%, confirming the dominant influence of benign mesh-related components. After exclusion (Figure 14b), a modest improvement is observed, most notably for tooth 17, with a TPCD of 82%. However, many teeth still yield values of 0–50%. The results for gear 1 are summarized in Figure A15.

The low TPCD of the SK-SE technology can be attributed to two main factors. First, the SK is fundamentally a second-order energy detector: SK-based filtering followed by a Hilbert demodulation and squaring amplifies both the fault-related and structural resonances. In practice, the structural effects remain after SK filtering due to: (i) the broad SK plateaus or lobes that pass resonances (e.g., the ~3 kHz band), and (ii) the finite STFT windowing and leakage. As a result, the structural resonances are boosted alongside the sparse impulsive events. Second, the fault-related impulses are subject to tooth-domain smearing. The timing variability in the tachometer, angular resampling, and natural fluctuations in impact onset the shift impulses by small fractions of a tooth, spreading their energy across neighbouring bins when the revolutions are aligned and averaged. Because the squared envelope amplifies the structural resonances as much as short impulses, this misalignment further blurs the fault signatures and increases the overlap between healthy and defected distributions. Consequently, even with the appropriate SK-based demodulation, the SK technology provides limited TPCD.

To address the limitations of the squared envelope, this study introduces the integrated spectral kurtosis (ISK) as an effective diagnostic technology for gearbox fault diagnosis. Unlike the SE, which amplifies both fault-related and benign amplitude modulations, the ISK focuses on impulsiveness by summing only the SK values that exceed a threshold.

For each gear rotation period, the ISK is estimated by summing all threshold-exceeding SK values across frequency bins, yielding a scalar feature that reflects the total impulsive content within a cycle. This eliminates the need for narrowband selection or a Hilbert demodulation, while enhancing the sensitivity to weak or localized faults that might otherwise be masked. The diagnostic effectiveness is evaluated by estimating the total probability of correct diagnostics (TPCD).

**Figure 15:**
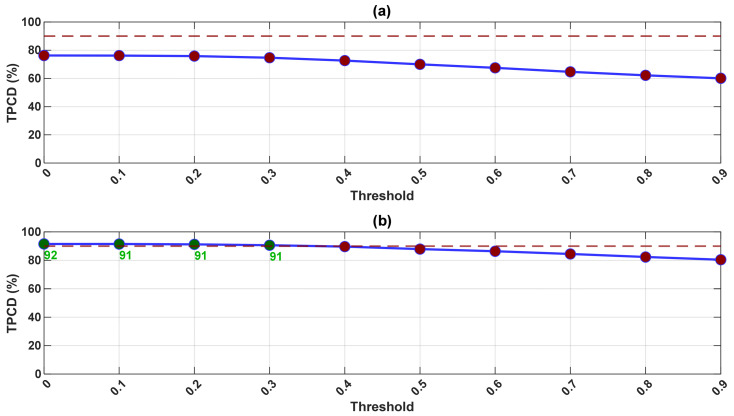
Variation in the total probability of correct diagnosis (TPCD) as a function of the spectral kurtosis threshold used in the ISK estimation for pinion 1 before and after excluding the 3 kHz band: (**a**) before exclusion, (**b**) after exclusion. The red dashed line indicates the 90% TPCD threshold; values above this threshold correspond to effective diagnosis. Coloured dots show TPCD values (green: effective, red: non-effective), and green numbers indicate TPCD for effective cases only.

The SK threshold, which defines the minimum value included in the summation, is varied systematically from 0 to 0.9. For each threshold, the ISK is estimated per realization and used to estimate the TPCD. Figure 15 illustrates the variation in the TPCD with the threshold for pinion 1 before (Figure 15a) and after (Figure 15b) exclusion of the 3 kHz band.

**Figure A16:**
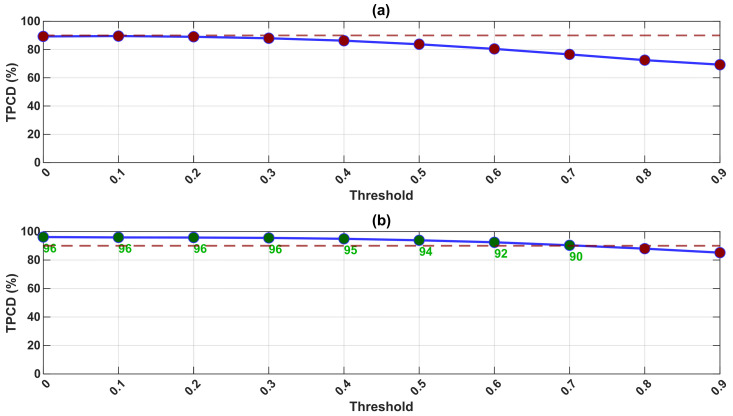
Variation in the total probability of correct diagnosis (TPCD) as a function of the spectral kurtosis threshold used in the ISK estimation for gear 1, before and after excluding the 3 kHz band: (**a**) before exclusion, (**b**) after exclusion. The red dashed line indicates the 90% TPCD threshold; values above this threshold correspond to effective diagnosis. Coloured dots show TPCD values (green: effective, red: non-effective), and green numbers indicate TPCD for effective cases only.

Prior to the band’s exclusion, the TPCD values remain low across all thresholds, with only a small peak at 0.0–0.3. This reflects the influence of structural resonances, particularly the persistent 3 kHz band. After the 3 kHz band’s exclusion, the TPCD increases sharply at low thresholds, reaching 91–92%. At thresholds above 0.5, the TPCD declines, indicating that critical impulsive information is discarded only when the highest SK peaks are retained. Comparable trends are observed for gear 1 (Figure A16).

The observed dependency of the TPCD on the threshold provides insight into the nature of gearbox fault signatures. Lower threshold values preserve a larger portion of the impulsive background associated with a fault, while higher thresholds retain only high-amplitude events. Once the threshold exceeds a certain level, diagnostically relevant information is progressively discarded, leading to a decrease in the TPCD.

The low TPCD of the ISK technology in the presence of structural resonance 3 kHz band suggests that, although some impulse activities are detected, particularly around 9 kHz and 18 kHz, their impulsive energy is not sufficient to provide an effective fault diagnosis. Given that both pinion 2 and gear 2 were confirmed to be healthy during inspection, the frequency components are propagated from defects in pinion 1 and gear 1, rather than originating from pinion 2 and gear 2. At higher thresholds, the TPCD drops further, confirming that subtle transient contributions are lost when only the strongest SK peaks are retained.

The TPCD of the ISK technology is evaluated before (version 1) and after (version 2) excluding the 3 kHz band. Across pinion 1 and gear 1, version 2 consistently delivers higher TPCD values than version 1. For pinion 1, the TPCD increases from 76.24% to 91.53%, with Fisher’s exact test [46] confirming the improvement to be statistically significant (*p* = 3.39 × 10^−196^). Comparable gains are observed for gear 1, where version 2 also achieves significantly better separability (*p* = 2.04 × 10^−39^).

The diagnostic effectiveness of the ISK technology is further evaluated by comparing the probability of incorrect diagnosis between version 1 (before excluding the 3 kHz band) and version 2 (after band exclusion). As summarized in Table 1, the gain is quantified as the ratio of error probabilities between the two versions. For pinion 1, the incorrect diagnosis rate decreases from 0.24% (version 1) to 0.08% (version 2), corresponding to a gain factor of 2.81. A similar improvement is observed for gear 1, where the error probability drops from 0.10% to 0.04%, yielding a gain of 2.70. These results confirm that excluding the 3 kHz band significantly improves the effectiveness of the ISK across all gear stages. Version 2 consistently achieves a lower incorrect diagnosis, demonstrating its superiority over version 1.

When comparing their diagnostic effectiveness, the ISK technology consistently outperforms the SK technology. Across all gear stages, the SK yields TPCD values below 90%, with limited separability between healthy/defected states. In contrast, the ISK achieves values exceeding 90%, reaching up to 96% for gear 1 at low thresholds.

In summary, the ISK provides a reliable, deployment-ready technology for gearbox diagnostics.

### 5.2. Motor Current Technology Results

The current and rotational speed signals are acquired at 40 min intervals, with each recording spanning 1200 s. To extract the spectral amplitudes associated with gear-related modulations, the envelope spectrum is estimated using a short-time chirp Fourier transform (STCFT). A 100 s external analysis window is applied with 50% overlap between successive segments to enhance the temporal resolution. The internal segments are tapered using a Hamming window to reduce the spectral leakage. The rotational speed is estimated from the tachometer pulses using the elapsed-time method [44].

The spectral amplitudes of the fundamental and harmonic frequencies of the first-stage gear mesh are extracted from the envelope spectrum as quantitative diagnostic features. The total probability of correct diagnosis (TPCD) is estimated, with a diagnostic threshold of 90%. Only the harmonics exceeding this threshold are retained. The harmonics exceeding the 90% TPCD threshold are summarized in Table 2. These include the stage 1 (pinion 1 and gear 1) second and fourth harmonics. All other harmonics are excluded due to their low TPCD.

The TPCD of vibration-based and current-based technologies is compared to assess their relative effectiveness in gearbox fault diagnosis. The vibration analysis employes the integrated spectral kurtosis (ISK) technology, targeting the impulsive transients and providing the most reliable results by summing only the threshold-exceeding SK values. The ISK technology achieves a TPCD of 96.1%, clearly outperforming the current-based technology, which only reaches 90%. Fisher’s exact test confirms this difference to be statistically significant (*p* = 7.32 × 10^−49^), establishing the superiority of the ISK technology. The extremely low *p*-values obtained confirm that the observed TPDC differences are statistically significant and unlikely to arise from random variation, thereby reinforcing the effectiveness of the proposed ISK technology.

Although the MCSA based technology demonstrates reasonable diagnostic effectiveness for gear 1, its TPCD remains lower than that of the ISK technology. Nevertheless, the non-intrusive nature of MCSA makes it attractive for certain industrial applications. Table 3 summarizes the TPCD of the proposed ISK technology in comparison with the conventional SK squared envelope and MCSA technologies.

## 6. Conclusions and Future Work

This study proposes and investigates a novel integrated spectral kurtosis (ISK) technology for fault diagnostics and compares it with the traditional vibration spectral kurtosis (SK)-based technology and motor current signature analysis technology for gearbox fault diagnosis. The proposed integrated spectral kurtosis (ISK) technology is defined as a summation of threshold-exceeding SK values across the frequency range.

The main results of the comprehensive experimental trials are as follows:

- For vibration gearbox diagnostics, the ISK technology outperforms the SK technology by integrating only the threshold-exceeding SK values. For pinion 1 the diagnostics has a 91.5% TPCD, and for gear 1 the diagnostics has a 96.1% TPCD. The traditional SK technology remains at a maximum 80% TPCD, confirming its limited diagnostic capability. The gains in the total error probability of diagnosis are 2.4 times and 5.1 times.
- For motor current diagnostics, for gear 1 the diagnostics shows borderline separability, reaching a maximum 90% TPCD, which is lower than the 96.1% TPCD using the ISK technology (Fisher’s exact test criterion is *p* = 7.32 × 10^−49^). The gain in the total error probability of diagnosis is 2.6 times.
- These diagnosis results will open the door for the wide industrial implementation of the novel ISK technology for various rotating machinery.
- The integrated spectral kurtosis (ISK) technology achieves a higher TPCD compared to the SK vibration technology and the MCSA technology.
- The results of this study are essential for fault diagnosis. The proposed ISK technology presents a novel conceptualization and will make a considerable impact on fault diagnosis in electrical and mechanical engineering via motor current signature analysis, vibration analysis, ultrasound analysis, etc.
- Despite the effective diagnosis results, this study is comprehensively validated only for the tested industrial gearbox under typical operating conditions.

The ISK technology provides a novel effective quantification of impulsive content across the whole frequency range without SK filtering and envelope-based processing. This allows for the more effective diagnosis of machinery fault because there is no loss of any essential diagnosis information. This essentially advances fault diagnosis in multiple practical cases in which fault-related information is not confined to specific SK-selected frequency bands.

The applicability of the proposed ISK technology to other gearbox designs and other rotating machinery remains to be further investigated.

## Figures and Tables

**Table 1:** Diagnostic performance of the ISK before (version 1) and after (version 2) 3 kHz exclusion.

Component	Incorrect (Version 1)	Incorrect (Version 2)	Gain
Pinion 1	0.24	0.08	2.81
Gear 1	0.1	0.04	2.7

**Table 2:** The TPCD of individual mesh harmonics.

Component	1st Harmonic	2nd Harmonic	3rd Harmonic	4th Harmonic
First stage	<90%	90%	<90%	90%

**Table 3:** Comparative diagnostic effectiveness for gearbox fault diagnosis.

Diagnostic Technology	Signal Type	Diagnostic Feature	Pinion 1 TPCD (%)	Gear 1 TPCD (%)
Integrated Spectral Kurtosis (ISK)	Vibration	Integrated threshold-exceeding SK (scalar)	92.0	96.1
Conventional SK Squared Envelope (SK-SE)	Vibration	SK-based squared envelope	≤80.0	≤80.0
Motor Current Signature Analysis (MCSA)	Stator current	Unnormalized mesh harmonic amplitude (UMHA)	90.0	

## Data Availability

The data presented in this study are not publicly available due to confidentiality and industrial restrictions.
